# A novel arthroscopically assisted reduction technique for three patterns of posterolateral tibial plateau fractures

**DOI:** 10.1186/s13018-020-01901-5

**Published:** 2020-09-03

**Authors:** Yang Yang, Xiaoxiao Zhou, Houlin Ji, Xiaobo Zhou, Linchao Ye, Mengqin Zhang

**Affiliations:** 1 Department of Orthopedics, Taizhou Hospital of Zhejiang Province Affiliated to Wenzhou Medical University, Linhai, Zhejiang China; 2 Department of Orthopedics, Shanghai University of Medicine & Health Sciences Affiliated Zhoupu Hospital, Shanghai, China; 3 Graduate School of Shanghai University of Traditional Chinese Medicine, Shanghai, China; 4 Intensive Care Unit, Taizhou Hospital of Zhejiang Province Affiliated to Wenzhou Medical University, 150 Ximen Street, Linhai, 317000 Zhejiang Province China

**Keywords:** Posterolateral tibial plateau fracture, Arthroscopically assisted reduction and fixation, Fracture patterns, Restoration, Soft tissue injury

## Abstract

**Background:**

Posterolateral tibial plateau fractures (PTPF) remain a challenge for orthopedics surgeons because the special anatomical structures of the posterolateral corner of knee joint including the fibular head, the lateral collateral ligament, and the peroneal nerve, which impedes the exposure of the fracture fragments and need irregular implants to get a stable fixation. The purpose of present study was to introduce a new articular fracture fragments restoration technique for three patterns of PTPF and investigate the relationship between associated soft injuries and fracture patterns.

**Methods:**

From May 2016 to April 2018, 31 patients with PTPF who had undertaken arthroscopically assisted reduction and fixation (AARF) were enrolled in present study. Demographic data, pre-operation, and post-operation X plan films, three-dimensional computed tomography (CT) scans and magnetic resonance imaging (MRI) were reviewed. Present samples were divided into three patterns with lateral inclination (LI), posterior inclination (PI), and parallel compression (PC) according to the orientation of the articular fragment inclination. Rasmussen anatomical score was used to assess the radiological results. Rasmussen functional score, Hospital for Special Surgery knee-rating Score (HSS), and range of motion (ROM) of the knee joint at the final follow-up were measured to evaluate the clinical outcomes.

**Results:**

In this series, the post-operation tibial plateau angle (TPA) was 9.7° ± 3.5°(range 4.0°–15.8°) and the Rasmussen anatomical score was 17.7 ± 0.7(range 16–18); clinical outcomes showed that the HSS score was 92.7 ± 21.8 (range 90–96) and the Rasmussen functional score was 27.9 ± 1.0 (range 26–30). Of all the patients, the anterior cruciate ligament (ACL) injuries including the ACL tibial attachment ruptures occurred in 16 patients (51.6%), meniscus lesions happened in 19 patients (61.3%), medial collateral ligament (MCL) injuries were founded in 13 patients (41.9%). The number of ACL injuries including the ACL tibial attachment ruptures in the PI fracture pattern (12 cases) is significantly higher than LI (2 cases) and PC (2 cases) fracture pattern (*p* < 0.05).

**Conclusion:**

Profound understanding the different patterns of PTPF and using our reduction technique will facilitate to restore the main articular fracture fragments. The PI fracture patterns have a significant high incidence of the ACL ruptures.

**Level of evidence:**

Therapeutic study, Level IV.

## Introduction

Posterolateral tibial plateau fracture (PTPF) is an intraarticular fracture with a low incidence in the past decades [1–3], while the prevalence of PTPF increased dramatically in China because the electric scooter widely used in public [4]. The optimal treatment of PTPF consists of articulation congruency, rigid fixation, periarticular soft tissues repair, which will achieve joint stability, enable the earlies mobilization, and restrain the development of degenerative osteoarthritis. Open reduction and internal fixation (ORIF) via different approaches has been reported in literatures with satisfactory clinical outcomes [1, 3, 5–8]. However, PTPF remains a challenge for orthopedics surgeons because the special anatomical structures of the posterolateral corner of knee joint including the fibular head, the lateral collateral ligament, and the peroneal nerve, which impedes the exposure of the fracture fragments and need irregular implants to get a stable fixation.

AARF has been applied for tibial plateau fractures since 1985 with advantages of direct visualization of the articular surfaces, anatomical reduction of the fracture fragments, minimal invasions, and the possibility of assessing and treating the periarticular soft tissue injuries [9, 10]. AARF has been conducted in almost all kinds of tibial plateau fractures patterns and excellent clinical and radiological outcomes have been showed in literatures, especially for the treatment of Schatzker’s I–III fractures [11–15]. Several techniques have been reported to elevate the articular surface to get an anatomical restoration, and the metal bone tamp is the most used technique in practice. While the restoration of the articular surface fracture fragments remains a time-consuming procedure with a single metal bone tamp. Therefore, a profound recognition of the fracture patterns of PTPF may facilitate the restoration procedures with the metal bone tamp.

Schatzker’s classification was proposed on the basis of anteroposterior plan films of the knee joint [16, 17], which makes it difficult to recognize the posterolateral plateau fractures of the tibial. Luo’s three column classification was developed according to the CT scans, which reveals the posterior column of the tibial plateau [18]. But it has limited facilitations to guide the practice of the AARF for PTPF. High incidence of periarticular soft tissue injuries associated with tibial plateau fractures has been reported in literatures [19–21]. However, the relationship between periarticular soft tissues injuries and the tibial plateau fracture type has not been investigated. Hence, the purpose of present study was to evaluate the clinical and radiological outcomes of our new restoration technique for different fracture patterns of PTPF and investigate the relationship between the associated soft injuries and fracture patterns of PTPF.

## Method

From May 2016 to April 2018, 33 consecutive patients who were diagnosed with closed PTPF had undertaken AARF. The inclusion criterions were (1) posterolateral plateau fracture confirmed by CT scans; (2) unilateral fracture; and (3) closed fractures. The exclusion criterions were (1) age < 18 years; (2) open fractures; (3) polytrauma; (4) patients who underwent conservative treatment or open reduction internal fixation; (5) pre-existing severe degenerative joint disease. Two patients lost final follow-up, and 31 patients were included in present study finally.

The sample consisted of 13 males and 18 females with 16 left limb injuries and 15 right limb injuries. The mean age at surgery was 41.1 ± 13.0 years (range 19–65 years), and the mean hospital stay was 13.4 ± 4.3 days (range 6–24 days). The average follow-up was 24.9 ± 6.6 months (range 18–36 months). A total of 14 patients (45.2%) were injured by electric scooters or bicycles accidents, 10 patients (32.3%) were injured as the result of vehicle accidents, and 7 patients (22.6%) were caused by pedestrian fall.

### Radiological and clinical assessment

**Fig. 1:**
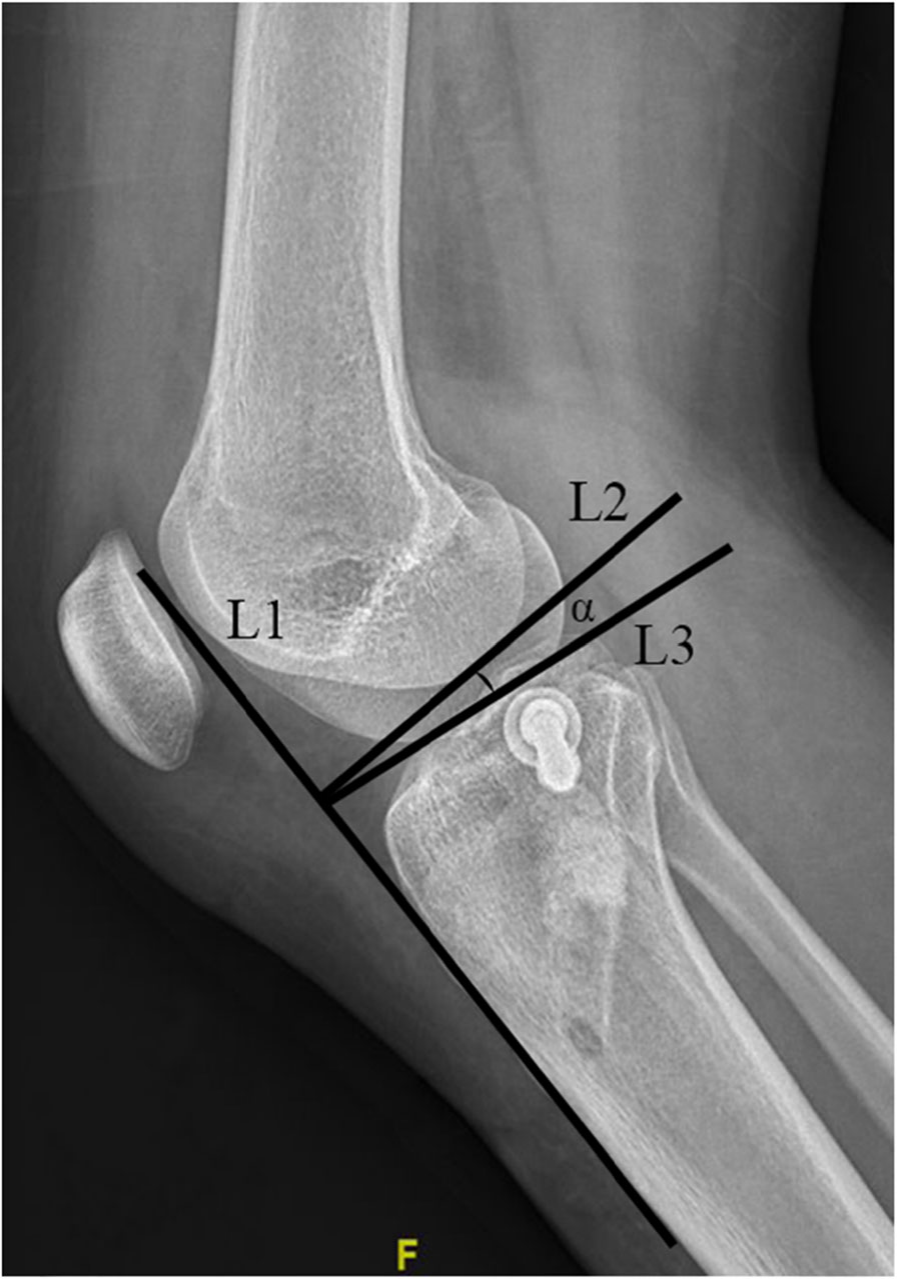
The measurement of TPA. L1 is parallel to the anterior crest of tibial; L2 is the perpendicular line of L1; L3 is the tangent line of the tibial plateau. Angle α represents the TPA

**Fig. 2:**
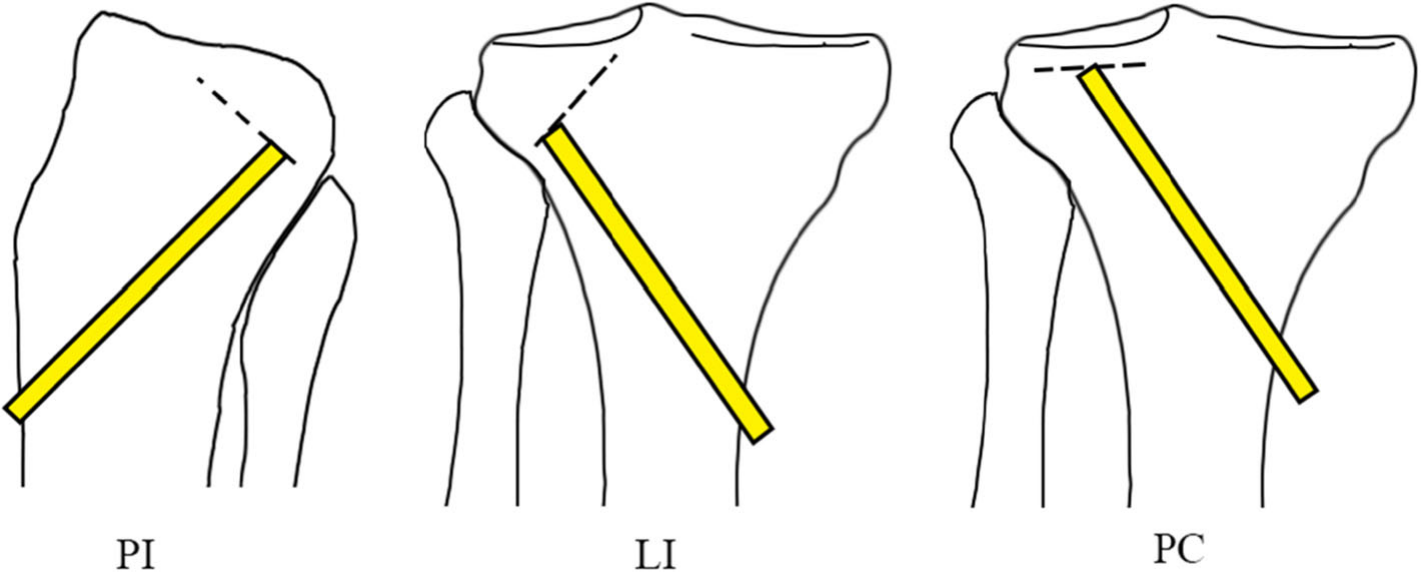
The three patterns of posterolateral tibial plateau fracture. The broken lines represent the inclination orientation of the main articular surface fracture fragments. PI, posterior inclination; LI, lateral inclination; PC, parallel compression. The yellow bar shows where the metal tamp placed

Demographic data, injury mechanisms were collected from patients’ records. Pre-operation and post-operation X plan films, three-dimensional CT scans as well as MRI of all patients were reviewed. Post-operation tibial plateau angles (TPA) were measured using Picture Archiving and Communication Systems (PACS) (Fig. 1). The orientation of the articular surface of the tibial was reviewed through three-dimensional CT scans. According to the orientation of the articular fragment inclination, we divided present samples into three patterns with lateral inclination (LI), posterior inclination (PI), and parallel compression (PC) (Fig. 2). Rasmussen anatomical score was used to assess the radiological results. Rasmussen functional score, Hospital for Special Surgery knee-rating Score (HSS), and range of motion (ROM) of the knee joint at the final follow-up were measured to evaluate the clinical outcomes.

The associated soft tissue injuries were checked by MRI and physical examination pre-operation and then confirmed intra-operation. Operations in present study were performed by senior authors.

### Surgical procedure

Detailed pre-operation physical examination was performed after patients under general anesthesia or intraspinal anesthesia to assess the associated ligament injuries. The supine position with 90° flexion of the knee joint for all the patients. A conventional anterolateral port and an anteromedial port were created for observation and manipulation, respectively. Saline, drove by gravity instead of a pump, was used to irrigate the knee joint. After evacuating the hematoma in the articulation, a shaver was utilized to remove the clots and some synovium to make the visibility sufficient enough. Then, intra-articular lesions cloud be inspected, including articular fracture fragments, articular surface lesions, ligaments, and menisci ruptures [22].

**Fig. 3:**
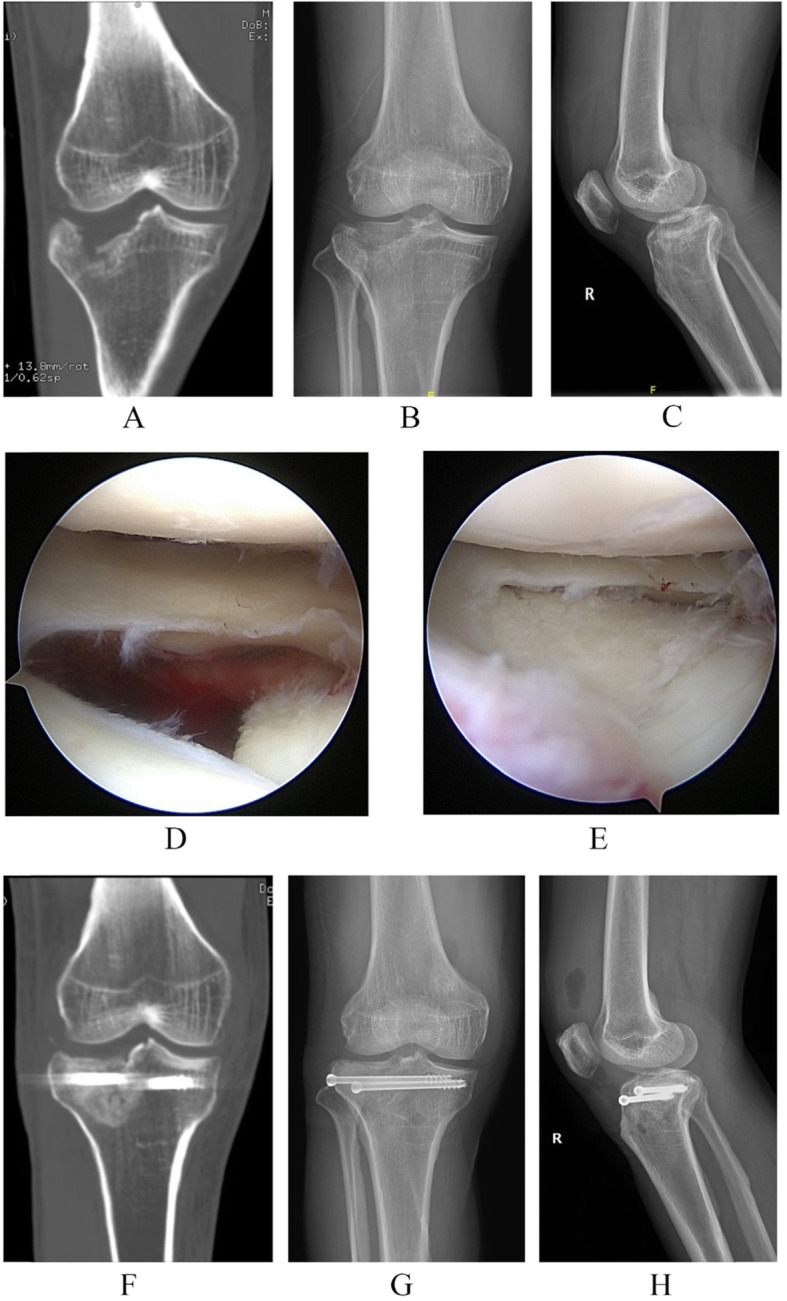
A 44-year-old male patient who was diagnosed with the lateral inclination type of posterolateral tibial plateau fracture with LI patterns. **a** The coronal image of three-dimensional CT scans shows the lateral inclination of the tibial fracture fragment. **b** The anteroposterior (AP) plain film of the injured knee. **c** The lateral plain film of the injured knee. **d** The tibial plateau fracture visualized with the assistance of arthroscopy. **e** After restoration of the fracture fragments with the surveillance of arthroscopy, the step-off was eliminated. **f** The coronal image of three-dimensional CT scans shows the restoration and the fixation. **g** The post-operation AP film of; H, the post-operation lateral plain film

The ACL guide was located at the lowest edge of the fracture fragments, that is, the ACL guide was set at the lateral edge for the LI pattern, placed at the posterior edge for the PI pattern and centered for the PC pattern. Then, a tunnel was drilled with a guide pin, and the tamp was introduced into this tunnel to lift the articular surface. One or two 2.0 mm k-wires were used to provide provisional fixation when articular surface congruence acquired. The calcium sulfate bone graft substitute (Biocomposites, UK) was used to fill the bony defect. The fracture fragments in this series were all elevated by metal tamp. Cannulated screws (Canwell, China) were used for the fixation of the PTPF without lateral cortical fracture of the tibial plateau, and lateral locking plates of tibial plateau (Canwell, China) were applied to fix the PTPF with lateral cortical fracture through the conventional anterolateral approach (Fig. 3). The meniscoplasty or meniscus repair were performed accordingly. MCL ruptures were repaired using traditional methods. The avulsion fracture of the tibial attachment of ACL was fixed with 2-0 Ethibond (Johnson & Johnson, USA) via conventional technique. The ligament reconstruction of ACL ruptures was performed secondary to fracture healing.

### Post-operative management

A standardized post-operative rehabilitation training program was conducted for all the patients. All patients received the continuous passive motion of the injured knee at the first day after surgery and the ROM was limited to 60° in flexion and 0° in extension. The ROM of passive motion was increased gradually. No weight-bearing activities were allowed in the first 4 weeks, partial weight-bearing activities were encouraged in the 4 to 8 weeks post-operation, full weight-bearing could be resumed at the 8 weeks after surgery accordingly.

### Statistical analysis

Statistical analysis was performed by SPSS 21.0 (SPSS Inc., Chicago, IL, USA). Statistical analysis was conducted with the use of a Fisher’s exact test for discrete variables. One-way ANOVA test was used to compare numeric variables, and Bonferroni test was applied to perform pairwise comparison. The continuous descriptive variables were present as mean ± standard deviation. Level of significance was set at 0.05 for all analyses.

## Results

In this series, the post-operation TPA was 9.7°±3.5°(range 4.0°–15.8°) and the Rasmussen anatomical score was 17.7 ± 0.7 (range 16–18); clinical outcomes showed that the HSS score was 92.7 ± 21.8 (range 90–96) and the Rasmussen functional score was 27.9 ± 1.0 (range 26–30) at the final follow-up. All patients achieved good or excellent Rasmussen anatomical score, Rasmussen functional score as well as HSS score. The range of motion(ROM) at the final follow-up were 2.7° ± 2.5°(range 0°–5°) in extension and 129.7° ± 10.8°(110°–150°) in flection. No significant difference was found in the number of patients, age, sex, injury side, hospitalization time, and causes of injury among three patterns (Table 1).

**Table 1:** General data of all patients

	LI	PI	PC
No. of patients	10	14	7
Mean age, years(range)	43.8 ± 15.7(24–62)	42.1 ± 9.6(23–58)	35.3 ± 15.0(19–55)
Sex, male/female	5/5	4/10	4/3
Injury side, left/right	3/7	9/5	4/3
Hospitalization time, days (range)	13.2 ± 2.7(10–19)	13.5 ± 5.6(6–24)	13.4 ± 3.7(10–21)
Causes of injury			
Electric scooter or bicycle accident	3	9	2
Vehicle accident	5	3	2
Pedestrian accidents	2	2	3

*LI* lateral inclination, *PI* posterior inclination, *PC* parallel compression

Of all the patients, the ACL injuries including the ACL tibial attachment ruptures occurred in 16 patients (51.6%), meniscus lesions happened in 19 patients (61.3%), MCL injuries were found in 13 patients (41.9%). The fibular head fracture and the lateral cortical bone of tibial plateau fracture could be observed in 5 patients (16.1%) and 13 patients (41.9%), respectively.

The number of ACL injuries including the ACL tibial attachment ruptures in the PI pattern (12 cases) is obviously higher than LI pattern (2 cases) and PC pattern (2 cases) (*p* < 0.05); while there is no significant difference in the incidence of the MCL lesions, meniscal injuries, lateral cortical bone fractures of the tibial plateau, and fibular head fractures between three fracture patterns (*p* > 0.05) (Table 2). The radiological and clinical results between the LI, PI, and PC patterns show no significant difference (*p* > 0.05) (Table 3). No posterolateral instability of the knee joint occurred.

**Table 2:** Relationship between fracture types and associated injuries

	ACL	MCL	Meniscus	LCB	FHF
	Injured/none	Injured/none	Injured/none	Fractured/none	Injured/none
LI	2/8	7/3	9/1	4/1	4/6
PI	12/2*	4/10	7/7	3/7	1/13
PC	2/5	2/5	3/4	0/2	0/7

*p < 0.05, compared with LI and PC group

*ACL* anterior cruciate ligament, *MCL* medial collateral ligament, *LCB* lateral cortical bone, *FHF* fibular head fracture, *LI* lateral inclination, *PI* posterior inclination, *PC* parallel compression

**Table 3:** Radiological measurements and clinical outcomes of different fracture types

	TPA	Rasmussen anatomical score	ROM		Rasmussen functional score	HSS
			Flection (°)	Extension (°)		
LI	8.1 ± 1.9(4.9–11.0)	17.6 ± 0.8(16–18)	132 ± 14.8(110–150)	4.0 ± 2.3(0–5)	27.2 ± 0.6(26–28)	91.7 ± 1.4(90–94)
PI	10.8 ± 4.3(4.0–15.8)	17.7 ± 0.7(16–18)	132 ± 9.2(120–145)	2.5 ± 2.6(0–5)	28.4 ± 1.2(27–30)	92.9 ± 2.0(90–96)
PC	10.0 ± 2.9(5.3–13.0)	17.7 ± 0.8(16–18)	130 ± 14.1(120–140)	2.5 ± 3.5(0–5)	28.0 ± 0.8(27–29)	93.6 ± 1.8(90–95)

*p < 0.05, compared with PI and PC group

#*p* < 0.05, compared with PI group

*LI* lateral inclination, *PI* posterior inclination, *PC* parallel compression, *TPA* tibial plateau angle, *ROM* range of motion, *HSS* Hospital for Special Surgery knee-rating Score

## Discussion

Arthroscopy has been used for the treatment of tibial fractures for several decades. However, it is difficult and time-consuming to manage the articular fracture fragments using a single tamp under arthroscopy because the relative-free motion of fracture fragments and the small tip of the metal tamp cannot hold the fracture fragments.

To circumvent this problem, an inflatable bone tamp was used to restore the depressed articular fracture fragments [23]. While the restoration was achieved by inflating the balloon which was introduced into the cancellous bone cavity, and the inflated balloon may lead to or deteriorate the lateral cortical fracture of tibial plateau. It was hard to control the force direction to restore the fragments; a metal tamp was still needed in the operation to get an anatomical restoration. Moreover, the high medical cost impeded the popularization of this technique. Instead of inserting the metal bone tamp beneath the center point of the main fracture fragments regardless of the fracture patterns of PTPF, we divided PTPF into three patterns basing on the inclination of the main articular surface. Thus, we set the metal tamp under the lowest edge of the fracture fragments to elevate the articular surface (Fig. 2), which can make the movement of the fracture fragment look like closing a door. Therefore, the fracture fragment turns over from high edge and the tilted restore with one side less restoration and the other side over restoration could be avoided.

In present study, all patients had excellent or good Rasmussen anatomical scores, Rasmussen functional scores, and HSS score at final follow-up. Kayali et al. performed the AARF for Schatzker type I to III tibial plateau fractures. The clinical outcomes of 21 patients after a mean follow-up of 38 months showed that 62% patients got an excellent Rasmussen functional score and 28% achieved a good score. Most patients had satisfactory radiological results with 52% excellent and 33% good Rasmussen radiological score [15]. Chiu et al. reported AARF could be managed for Schatzker type IV to VI tibial plateau fractures. In his series, after an average follow-up of 86 months (range 60 to 108 months), the mean Rasmussen functional score was 25.9 with 11(44%) patients had excellent score, 12 (48%) patients got a good result, 2(8%) patients achieved a fair outcome, the mean Rasmussen radiological score was 15.8 [24]. Marco et al. compared the clinical and radiological results between AARF and ORIF for the treatment of Schatzker type I–III tibial plateau fractures with a mean follow-up of 44.4 months. The outcomes revealed that arthroscopy-assisted reduction and fixation for type I–III tibial plateau fractures could provide a better clinical outcomes [25]. In present study, all patients were classified as Schatzker type I–III, and the three-dimensional CT scans confirmed the PTPF. The clinical outcome with a mean follow-up of 24.9 ± 6.6 months was better than previous studies as all patients got a good or excellent Rasmussen functional score and HSS score, which was benefited from the anatomical restoration and the early motion of knee joint acquired by present restoration technique. While the rigidness of fixation of the fracture fragments is always concerned by many surgeons.

In present study, we fulfilled the subarticular metaphyseal cavity with the calcium sulfate bone graft substitute, which was easy to be delivered into the defects. Other important merits of calcium sulfate bone graft substitute were osteoconductive abilities, bioabsorbable properties, and the natural bone similar structures which can provide a stronger support of the articular fracture fragments than autogenous and allograft bones. With the purpose of providing a stable fixation for PTPF with lateral cortical bone fractures, a lateral locking plate of tibial plateau was implanted, and 3 or 4 screws of the transverse arm were inserted. The plate should be placed more posteriorly so that the at least 1 or more screws could fix the PTPF, while one or two cannulated screws for the PTPF without lateral cortical bone fractures fixation was stable enough for early motion of knee joint and could minimize the incision. Therefore, the stable fixation of the fragment could be achieved by the strong support of the filled calcium sulfate bone graft substitute and the rigid fixation of screws or plates. The mean TPA at the final follow-up was 9.7° ± 3.5° compared to the normal northeast Chinese lateral TPA of 8.44° ± 2.76°, and no posterolateral instability of the knee joint was found, which implies that the fixation was rigid enough, and no significant subsidence of the articular fracture fragments happened after a full weight-bearing activities [26].

The high prevalence of associated soft tissue lesions of tibial plateau fractures has been noticed as the utilization of arthroscopy and MRI [20, 21]. In present study, we also found a high incidence of associated soft tissue injuries in tibial plateau fractures, even just PTPFs were investigated. ACL injuries and meniscal lesions were the most commonly concomitant soft tissue injuries in our study. Gardner et al. reported a 99% incidence of concomitant soft tissue injury in 103 fractures [27]. An arthroscopic evaluation of associated soft tissue lesions in tibial plateau fractures of 98 cases performed by Mohamed revealed that the rate of associated soft tissue lesions as high as 71% [20]. However, the relationship between soft injury sites and the tibial fracture patterns has not been investigated to date. Our results showed that a significant high rate of ACL ruptures occurred in the PI fracture pattern compared with the LI and the PC fracture pattern. No significant difference was found among three fracture patterns regarding of meniscal lesions, MCL injuries, lateral cortical bone fractures of tibial plateau and fibular head fractures.

Generally, the “roll-back” effect of the knee joint makes the lateral condyle of femur contacts to posterolateral tibial plateau when the knee articulation is in flexion position, then a valgus and axial loading is acting on the contacting surface of tibial plateau and leads to PTPF [28–30]. While this mechanism cannot explain why the main articular fracture fragment has different inclination directions and what is the relationship between associated soft tissue injuries and the fracture patterns of PTPF.

When patients fall from bicycles or electric scooters, the knee joint was in flexion, the axial force come from the reactive force of the lateral femur condyle acted on the posterolateral tibial plateau results in PTPF. More exactly, the lateral femur condyle slid posterior along the slope of the lateral tibial plateau as the axial force sustained, which leads to PI fracture pattern and a significant increased the slope of the lateral tibial plateau, causing hypertension of the ACL, especially the anteromedial bundle. ACL acts as the primary restraint against anterior tibial gilding in the sagittal plane and it also acts as a secondary restraint against the internal rotation in axial stability. In the flexion of the knee joint, the stress force of anteromedial bundle was significantly higher than the posterolateral bundle, and the anteromedial bundle tighten and twist around the slackened posterolateral bundle [31]. The excessive tension of the ACL resulted in the lesions of ACL or disruption of ACL tibial insertion. Therefore, the flexion of knee joint, the translation of tibiofemoral articulation, the increased tibial plateau slope, and the anatomical characteristics of ACL all contributed to the ACL injury. A valgus force and an axial force acted on the posterolateral tibial plateau leading to the LI of the fracture fragments. Theoretically, the valgus force of the knee joint would cause MCL injuries, but we did not find the significant difference among three groups in present study. This maybe because of a small sample in this study, or maybe the subsidence of posterolateral plateau decreased the tension of MCL. The PC fracture pattern, similar to Schatzker’s type III, occurs in elderlies who have lower bone mineral density compared to young patients. Therefore, a low energy axial and valgus force could result in this type of fractures [32].

### Limitations

Some limitations still existed in this study. Firstly, a relatively small sample was enrolled in present study. Secondly, we did not analyze the relationship between tibial plateau slope and the ACL injury. Thirdly, present study is a retrospective without control group designed study. A randomized controlled clinical study with a large sample and long-term is necessary to determine the efficacy of our technique.

## Conclusions

This study introduced a new restoration technique for managing the articular fracture fragments of PTPF basing on the three fracture patterns. Profound understanding the mechanism of different patterns of PTPF and using our reduction technique will facilitate to restore the main articular fracture fragments with AARF. ACL injuries and meniscal lesions were the most commonly concomitant soft tissue injuries. The PI fracture patterns have significant high incidence of the ACL ruptures.

## Data Availability

Not applicable

## Abbreviations

PTPF: Posterolateral tibial plateau fracture
CT: Computed tomography
MRI: Magnetic resonance imaging
LI: Lateral inclination
PI: Posterior inclination
PC: Parallel compression
ACL: Anterior cruciate ligament
MCL: Medial collateral ligament
LCB: Lateral cortical bone
FHF: Fibular head fracture
TPA: Tibial plateau angle
ROM: Range of motion
HSS: Hospital for Special Surgery knee-rating Score
